# The Relation of Lupus to Tubercle

**Published:** 1890-12-13

**Authors:** 


### MEDICINE.

### THE RELATION OF LUPUS TO TUBERCLE.

Volkmann had some years ago suggested that lupus was but a somewhat specialised form of a cutaneous tuberculosis, and it is well known that it is the only other disease in which the tubercle bacillus of Koch has ever been observed, although in its reactions that of leprosy appears to be nearly allied. Krokievicz has confirmed this view of the nature of lupus by inoculations of the bacilli obtained from three cases into a number of rabbits, four of which afterwards developed general tuberculosis.

